# Population-weighted efficiency in transportation networks

**DOI:** 10.1038/srep26377

**Published:** 2016-05-27

**Authors:** Lei Dong, Ruiqi Li, Jiang Zhang, Zengru Di

**Affiliations:** 1 School of Architecture, Tsinghua University, Beijing, 100084, China; 2 School of Systems Science, Beijing Normal University, Beijing, 100875, China

## Abstract

Transportation efficiency is critical for the operation of cities and is attracting great attention worldwide. Improving the transportation efficiency can not only decrease energy consumption, reduce carbon emissions, but also accelerate people’s interactions, which will become more and more important for sustainable urban living. Generally, traffic conditions in less-developed countries are not so good due to the undeveloped economy and road networks, while this issue is rarely studied before, because traditional survey data in these areas are scarce. Nowadays, with the development of ubiquitous mobile phone data, we can explore the transportation efficiency in a new way. In this paper, based on users’ call detailed records (CDRs), we propose an indicator named population-weighted efficiency (PWE) to quantitatively measure the efficiency of the transportation networks. PWE can provide insights into transportation infrastructure development, according to which we identify dozens of inefficient routes at both the intra- and inter-city levels, which are verified by several ongoing construction projects in Senegal. In addition, we compare PWE with excess commuting indices, and the fitting result of PWE is better than excess commuting index, which also proves the validity of our method.

Almost all of the dynamical processes we concern within cities, such as human mobility^1^,^2^,^3^,^4^, epidemic spreading^5^,^6^,^7^,^8^, and socio-economic activities, are driven by population interactions and based on transportation networks. In a broad sense, almost all urban flows (e.g. population, products, energy, information) are carried by “transportation networks”. After realizing the importance, most cities are stretching their transportation networks to promote urban development, but it is too costly to build new routes to reach every part of a city. Thus, analyzing and improving the efficiency of transportation networks are of great importance, since the efficiency improvement can not only decrease energy consumption and CO_2_ emissions^9^, but also accelerate urban flows, which are critical for the sustainability and effective operation of cities^10^.

Previous researches on transportation efficiency mainly focus on three separate ways: one is about the topology of networks, which is devoted to a general network efficiency, which can be applied to information exchange and transportation by treating the inverse of distance between node pairs as local measure of efficiency between them^11^. Another is about the geometric properties of spatial networks^12^,^13^, and develops an indicator named *route factor* (denote as 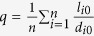) to measure the network efficiency by calculating the mean ratio of the distance along edges (*l*_*i*0_) and Euclidean straight line (*d*_*i*0_)^14^,^15^. The other one concentrates on the job-housing balance^16^ or excess commuting [i.e., the difference between the average actual commuting time (or distance) and the theoretical minimum average commuting time (or distance)]^17^,^18^. The first two do not consider the real population distribution, while the third one suffers from the use of statistically improbable variable (minimum average commuting distance) as a benchmark^18^, and is limited in finding local inefficient routes. Some recent work uses hypergraphs to study transferability of collective transportation line network and begins to concern with passenger system level^19^. In addition, these methods need either road network data (e.g. ordnance survey) or transportation survey data, which are costly and time consuming, and might not be available in less-developed countries. The data scarcity problem adds up many difficulties to the empirical analysis of transportation efficiency in these areas, where the traffic conditions are usually not so good due to the undeveloped economy and infrastructure.

Today, we leave tremendous “digital footprints” (geo-located data collected by electronic equipment) in cities due to the increasing penetration rate of mobile phones^20^, social media^21^, transportation cards^22^, and credit cards^23^, among others. These “digital footprints” give us a dynamical perspective of the ongoing phenomena in cities^24^,^25^, and provide us new data source to explore the transportation efficiency. Among these data, call detailed records (CDRs) of mobile phone users are considered to be of the highest quality to estimate the population/traffic dynamics due to the high penetration rate and lower usage bias than social media data.

In this paper, based on mobile phone CDRs and other open accessed data, we overcome the above-mentioned shortcomings and develop a new indicator called population-weighted efficiency (PWE) to quantitatively measure the road network performance. The population weight factor is based on home-work matrices, which will be our Origin-Destination (OD) matrices, extracted from CDRs, and the road network data are based on Google Map Application Programming Interface (API). The contributions of our work are as follows: First, the real population distribution is imported as the weight to the *route factor*. Cities with the same road network structure (*route factor*) but different population distribution may have different efficiencies, they can be captured by our method rather than the traditional way. Second, mobile phone data and Google Maps are used as proxy variables for population and road networks separately, they are quite useful for empirical research in African cities^26^,^27^,^28^,^29^, where the urbanization is fast but the population census are costly and not timely updated, and road network data of the OpenStreetMap (OSM)^30^ are sparse. Third, our method can be applied to detect inefficient routes and provide suggestions for infrastructure development and improvement. For example, route pairs with a high volume of commuters and abnormal low PWE would be considered as in need of improvement or reconstruction. As a case study, for Dakar, we identify the most inefficient route pairs at both intra- and inter-city levels, and our results are partially verified by several ongoing construction projects (e.g., the Dakar-Diamniadio highway^31^,^32^).

In addition, we study ten main cities in Senegal and compare their PWEs with the most commonly used excess commuting index, and find that there is a significantly correlation between PWE and the average commuting time, and the fitting result of PWE is better than excess commuting index, which further proves the validity of our method.

## Results

### Data description

Our analysis is based on anonymous mobile phone datasets of CDRs and text exchanges at antenna resolution between more than 9 million users of the Orange/Sonatel Group in Senegal from January 1, 2013, to December 31, 2013^33^. The data are preprocessed^33^ and provided by D4D Challenge organizers and are systematized into sub-datasets: dataset 1 includes the hourly voice and text exchange between mobile phone towers, and dataset 2 includes fine-grained anonymized individual mobility data at the antenna level of approximately 300,000 randomly sampled users for each fortnight. See ref. 33 for a full detailed description of the dataset, and more related works based on D4D data can be found in ref. 34.

**Figure 1:**
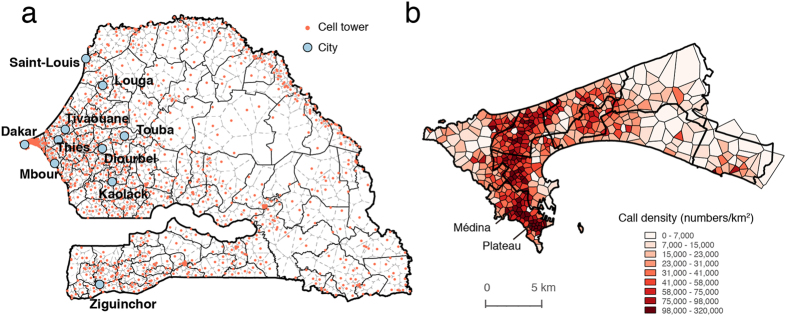
(**a**) Cell tower distribution (orange dots) for the ten main cities (blue dots) of Senegal. There are 1666 cell towers nationwide, 492 of which locate in Dakar. (**b**) The average daily call density map of Dakar; the Plateau and Médina are two of the most active regions in Dakar, which is also in agreement with the situation observed in reality. The gray dashed borders in (**a**) and thin black borders in (**b**) are Voronoi polygons generated according to cell towers. Maps were created using QGIS 2.8 (http://www.qgis.org).

We focus on ten main cities of Senegal, especially Dakar, which is the capital and the largest city in Senegal. The locations of the mobile phone towers and studied cities are displayed in Fig. 1a, and the averaged call density of Dakar is shown in Fig. 1b. According to the CDRs, we find that: i) on average, in each fortnight, there are more than 170,000 users within the region of Dakar, which accounts for almost 60% of the total sampled users (300,000); ii) Plateau and Médina are two of the most active regions during the daytime, which is also in agreement with the situation observed in reality. Instead of using the administration city boundary, we define the city boundary by the following criteria: i) divide the whole country into sub-regions by Voronoi tessellation, which are calculated according to the geographical coordinates of the antennas (see Fig. 1a); ii) calculate the population density of each cell based on WorldPop data^35^ and select all cells with a population density over 100 people/km^2^; and iii) combine the data with Google earth remote sensing data to filter out cells of non-built-up areas (artificial recognition). The first four columns of Table 1 show the names, population, and population density of the studied cities.

### Population-weighted efficiency (PWE)

**Figure 2:**
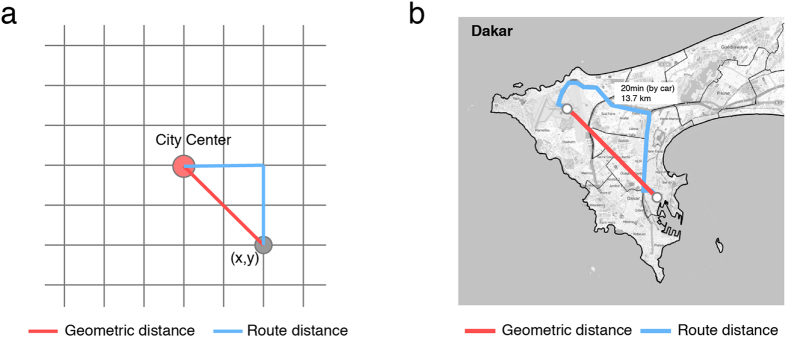
(**a**) The diagram of a monocentric city with Manhattan grids. The route distance from location (*x*, *y*) to the city center (0, 0) is |*x*| + |*y*|, and the geometric distance is (*x*^2^ + *y*^2^)^1/2^; (**b**) The geometric distance (red) and route distance (blue) between two points. The base map is from OpenStreetMap^30^ and modified in Illustrator.

If two places are geometrically close (with a small geometric distance, assigned as *d*_*e*_), but the route distance (*d*_*r*_) between them is large (Fig. 2a shows the difference between the geometric distance and route distance), then we can assume that this route’s structural efficiency *d*_*e*_/*d*_*r*_ is low and requires improvement. Here, the definition of structural efficiency is similar to the *route factor* proposed in refs 14,15. After repeating the process of calculating *d*_*e*_/*d*_*r*_ for every route, and average the results, we can get the efficiency of the whole road networks. This way weights each route equally, but in practice, the traffic volumes of routes are expected to be very different from one another, due to the uneven distribution of population. Therefore, if we weight the route factor by the proportion of population flows along each route, we can then obtain a more practical indicator, the population-weighted efficiency (PWE) for road networks, defined as:


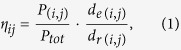


where *P*_(*i*,*j*)_ stands for the number of persons commuting from place *i* to *j* and 
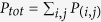
 is the total size of the population flows, *d*_*r*(*i*,*j*)_ is the route distance between *i* and *j*, and *d*_*e*(*i*,*j*)_ is the geometric distance. It is easy to calculate *d*_*e*(*i*,*j*)_ between any two locations according to their (longitude, latitude)s, and *d*_*r*(*i*,*j*)_ could be acquired from Google Map API (see the Methods, and Fig. 2b). By integrating the PWEs of each route pair, we can obtain the overall PWE of a city:


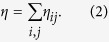


Based on our definition, *η* = 1 is the most efficient situation (there’s a direct route between any commuting pairs); the smaller *η* is, the less efficient it is.

### Users’ behavioral patterns and OD detection

To understand the PWE of a city, the first step is to identify the dynamic population distribution, *P*_*i*,*j*_, i.e., (at least) where people live and work, which is also called OD matrices and usually collected by field transportation survey. While for many developing or less-developed countries, the transportation survey is not available. Even for developed countries, the survey is usually conducted by the government once a year (or longer) for transportation planning purposes. Here, we use mobile phone call data to estimate the OD matrices, which has been widely accepted in academia^36^,^37^. However, unlike previous related works on OD analysis, which assume working hours according to practical experience^28^,^36^, we detect OD matrices by analyzing users’ behavioral patterns.

**Figure 3:**
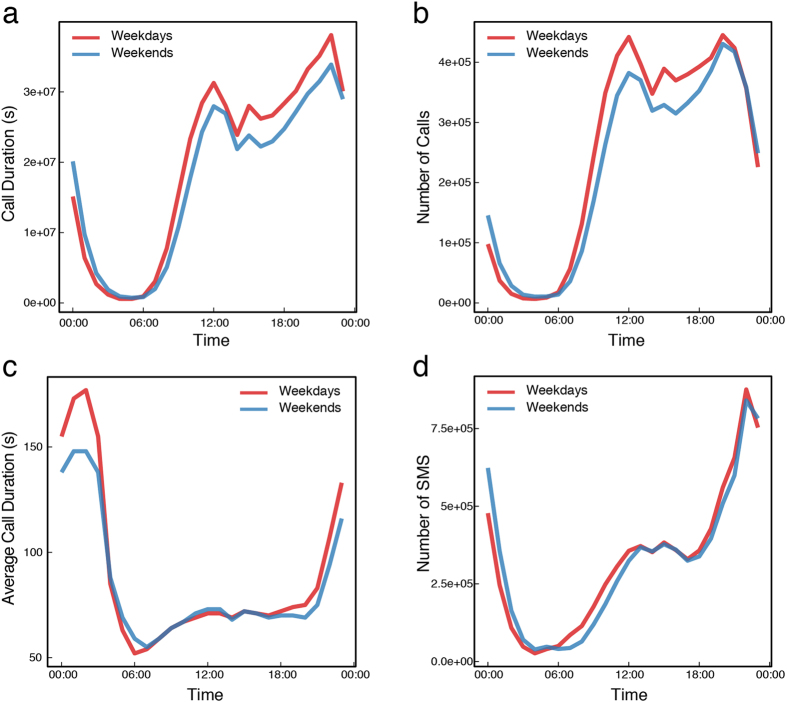
(**a**) The total call duration of all users; (**b**) the number of calls; (**c**) the average call duration; and (**d**) the number of SMS messages. We found the following results: (i) there are two peaks in calling activity (**a**,**b**): one is at noon (about 12:00) and another is at night (about 21:00–22:00); (ii) the average call duration during the day is about 60 seconds (**c**), which may be influenced by the charging mode or could just be a reflection of the daytime work character; (iii) people in Senegal are quite active during early night and they make longer calls compared to day time; few people make calls during the late night, but the ones who do, make much longer calls (**c**), and the number of short messages sent is very high at night (**d**).

For a closer look at the temporal patterns of the mobile phone calls, we find some common features of the calling patterns compared with cities in developed countries: there are two peaks in phone call activities, one at noon (about 12:00) and another at night (about 21:00–22:00, see Fig. 3a,b), and the second peak in Senegal is a little later than in most Spanish cities^25^. An attractive finding is that the average call duration during the day is about 60 seconds (Fig. 3c), which may be influenced by the charging mode or may be merely a reflection of the daytime work character, which is short, frequent and brief. Additionally, people in Senegal are quite active during nights: the call duration is long (see Fig. 3a), and the number of short messages is very high at night (especially late night, see Fig. 3d).

Based on these user behavioral patterns, we identify that the actual working hours are from 10:00 to 19:00. It is because the existence of a rapid increase in average call duration before and after this period, which corresponds to different active patterns. In Fig. 3d, we observe a striking peak in short messaging service (SMS) behavior at approximately 23:00, when the total call duration also peaks (see Fig. 3a,d), which implies another important behavior feature that usually occurs during leisure time (i.e., at home). Therefore, we set the home hours as 21:00 to 06:00 of the next day. For a user, we identify the most frequently visited location (tower) over the two periods mentioned above as his or her work/home places, respectively (see the Methods).

**Figure 4:**
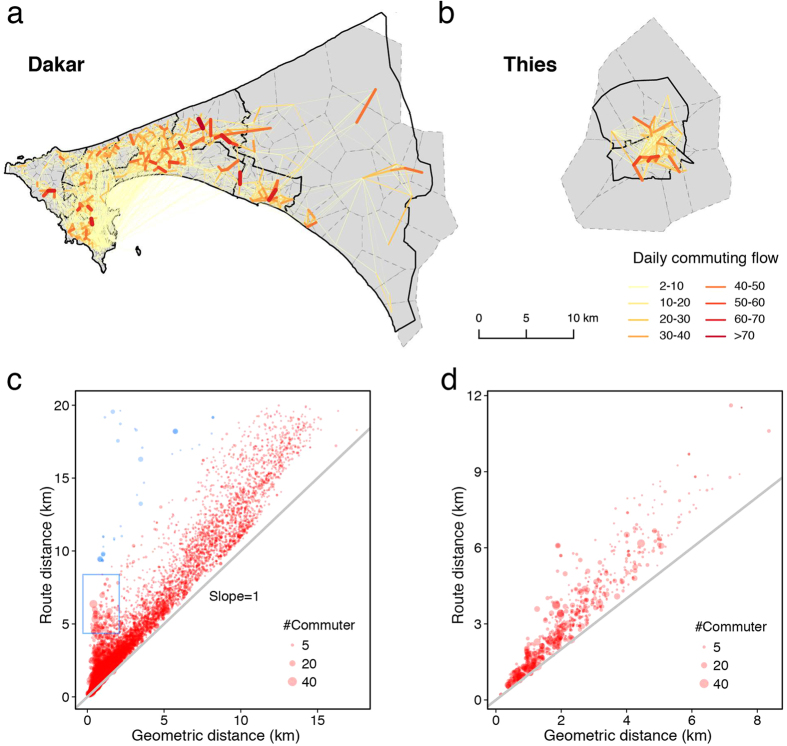
The daily OD pairs in Dakar (**a**) and Thies (**b**) extracted from the CDRs; Scatter plots of the route distance and geometric distance for Dakar (**c**) and Thies (**d**); the blue colored points and box indicate routes with large route distances and low geometric distances, which require improvement. The size of a node represents its OD volume, and the gray line has slope 1. Maps were created using QGIS 2.8 (http://www.qgis.org).

**Figure 5:**
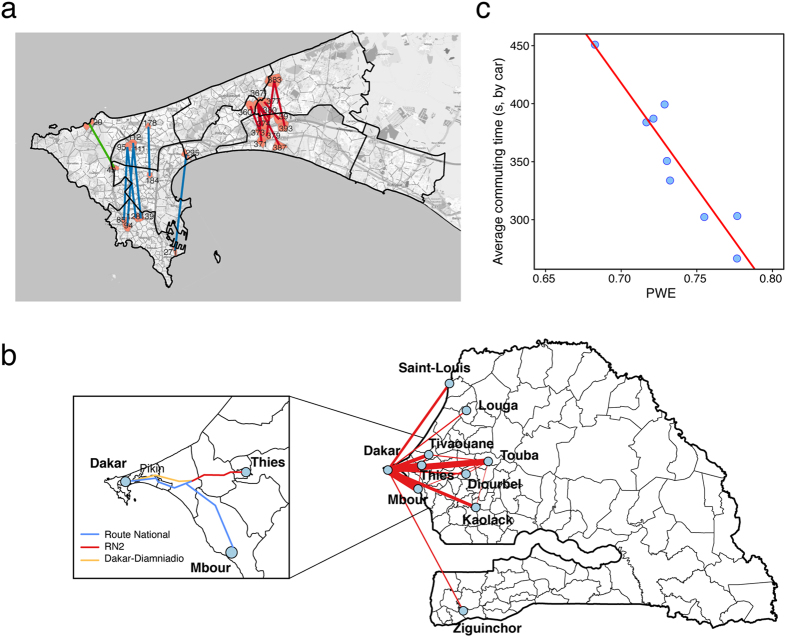
(**a**) The identified most inefficient route pairs in Dakar; there are three groups (red, blue, and green), and almost all are south-north trending. The base map is from OpenStreetMap^30^. (**b**) Commuting flows between ten cities, and the flows are aggregated from antenna resolution to city resolution. We find that there are a lot of people who commute between Dakar and its surrounding cities, especially Thies, Mbour, and Touba. (inset) The ongoing constructed Dakar-Diamniadio Highway (orange line), which makes transit faster between Dakar, Thies, and Mbour. (**c**) The scatter plot of the PWE (x axis) and average commuting time (y axis). The red line is the OLS linear regression fitting line, the coefficient is −1816 (244), the adjusted *R*^2^ is 0.87, and the p-value is 0.0001, which indicate a significant negative relationship between the PWE and average commuting time. Maps were created using QGIS 2.8 (http://www.qgis.org).

Therefore, we can extract an OD pair as the home to work place of each user at antenna resolution. By going through all of the users’ records, we obtain the OD matrices at both the intra- (see Fig. 4a,b and Supplementary Fig. S1, at antenna resolution) and inter-city levels (see Fig. 5b, at city levels, i.e., treat all the antennas within one city as a node). Figure 4 shows the OD matrices in Dakar (Fig. 4a) and Thies (Fig. 4b): it implies that a lot of people commute from west to east in Dakar, whereas the commuting pattern in Thies is from the center to the suburbs. It should be noted that these plots only show the geometric links between two places, rather than the real routes.

### PWE and its applications

After obtaining the OD matrices, we can query each OD pair’s locations to Google Map API, which can return the route distance and travel time between two locations. Figure 4c shows the scatter plots of route distance and geometric distance of Dakar and Fig. 4d exhibits Thies. The left-top points of Fig. 4c,d indicate routes with high route distances and low geometric distances, which implies that paths between these points are zigzags or complicated and of inefficiencies. The PWE of each road can be obtained by plugging the OD matrices and the ratio of the real route distance and the geometric distance into Eq. (1).

As an improved indicator for road networks efficiency, PWE has essentially three applications: i) detect inefficient routes within cities and ii) at inter-city level; iii) calculate overall commuting efficiency of each city. In the following, we will show the viability and effectiveness of the PWE.

First, as a case study in Dakar, according to the PWE of each road, we identify dozens of the most critical inefficient routes (see Fig. 5a, and Supplementary Table S1 gives the values of top ten inefficient routes). An attractive finding is that almost all of these critical inefficient routes are south-north trending, and can be divided into three groups (colored in red, blue and green separately in Fig. 5a). The existence of the red group in the east region may be mainly caused by the separation of the west-east trending highway, people are forced to detour the way from home to work. The blue group in the west region is mainly caused by the high weight of commuting volume, *P*_(*i*,*j*)_/*P*_*tot*_ and the detour. And the green group is another representative type that crosses an enclosed space (the airport in Dakar). The pattern of detected inefficient routes implies the necessity of constructing some fast transits between the north and south regions.

Second, PWE can be applied to inter-city level by considering the entire antennas within a city to be a node. Figure 5b shows the commuting flows between the ten main cities. We find that many people commute between Dakar and its surrounding cities, especially Thies, Mbour, and Touba, which indicates that the agglomeration degree of the urban population is very high in Senegal and that most of its population live near the Dakar region. With a similar calculation for PWE within the city, we can obtain the PWEs for city pairs. We find that there are several routes connecting Dakar, Thies and Mbour that are of the lowest PWEs, which indicates that we may need to build better roads between these city pairs. Our results are also partially verified by the ongoing construction of the Dakar-Diamniadio highway, which makes transit faster between Dakar, Thies, and Mbour^31^,^32^ (see inset of Fig. 5b).

**Figure 6:**
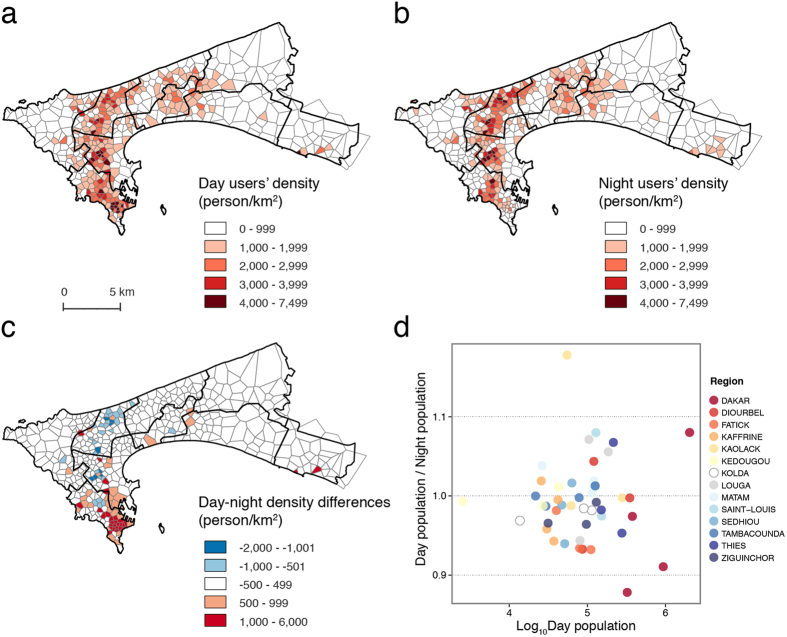
Day (**a**) and night (**b**) density maps of mobile phone users in Dakar, which could be an estimator of the population density; (**c**) the difference between the day and night density map (day minus night); red areas indicate that the day density is greater than the night density, and blue areas mean the opposite; (**d**) the scatter plot of the day-time population (x-axis in log scale) and the ratio of the day population over the night population (y-axis); each point represents one *Department*, and different colors represent different *Regions* (*Department* and *Region* are all Senegal administrative boundaries, and each *Region* includes 3–5 *Departments*). We find that Kaolack and Dakar are the two cities with the highest work-home separation degree. Maps were created using QGIS 2.8 (http://www.qgis.org).

Third, we compute the overall PWE for all ten main Senegalese cities (see the fourth column of Table 1), from which we find that Dakar is the most inefficient city with *η* = 0.683, whereas most city PWEs are about 0.73–0.78. The low efficiency of Dakar is mainly due to the lack of fast south-north trending transits and the high work-home separation, which could also be inferred from CDRs (see the Methods). In Dakar, We find that most of the users are distributed in Dakar Plateau, Grand Dakar, Médina, and Parcelles Assainies during the daytime (Fig. 6a), and Parcelles Assainies at night (Fig. 6b). Figure 6c depicts the work-home user differences in Dakar. For each department, we obtain a day/night population ratio, which evaluates the work-home separation situation (see Fig. 6d). We compare the separation degree of each *Region* and find that Dakar and Kaolack are the two *Regions* that have the highest separation degree (see Supplementary Table S2 for the calculation of the separation degree).

### Comparison between PWE and excess commuting index

There are many indicators could be used to measure commuting efficiency and job-housing balance in transportation literature^18^. In this section, we want to compare the most commonly used one, excess commuting, with PWE. First proposed by Hamilton^38^, the concept of excess commuting is used to measure the difference between the actual commuting distance (or time) and the theoretical minimum average commuting distance (or time). Mathematically, the equation of excess commuting could be expressed as follows:


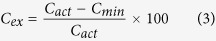


where *C*_*act*_ is the actual commuting distance, which is similar to *d*_*e*_ at individual level if measured by geometric distance, or *d*_*r*_ by route distance (see Supplementary Fig. S2). *C*_*min*_ is the minimum commuting distance when all working and living places are fixed in urban area, workers in a city choose work places that is closest to their living places. In other words, the assumptions of this transportation problem are: 1) the volume of origins, destinations of each area and the total flows between them are fixed; 2) individuals choose living and working places to minimize the total travel distance. The most optimal commuting flows could be derived by optimizing:


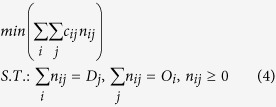


where *c*_*if*_ is the distance (or time) between area *i* and area *j*, *O*_*i*_ is the total number of people living in area *i*, and *D*_*j*_ is the total number of people working in area *j. n*_*ij*_ is the commuting flow from area *i* to *j*.

The minimum commuting flows (*n*_*ij*_) are calculated using *Scipy*.*optimize* package in *Python*, and the average minimum commuting distance (*C*_*min*_) is shown in the fifth column of Table 1. Since *C*_*act*_ varies for different definitions, here we calculate both geometric distance and route distance, and depict them in the sixth and seventh columns of Table 1 respectively. Obviously, *C*_*act*1_ (geometric distance) is smaller than *C*_*act*2_ (route distance). Combining with Eq. 3, we could get excess commuting indices, *C*_*ex*1_ and *C*_*ex*2_ (see Table 1).

To compare PWE and excess commuting, a third benchmark is needed. Here, we choose average commuting time, which is a direct reflection of the transportation efficiency. We calculate the average commuting time in each city (see the Methods), and find that both PWE and excess commuting indices highly correlate with average commuting time (by OLS regression), which indicates both of them could be used to measure the efficiency of a city, however, PWE is better because its correlation coefficient is much higher (*R*^2^ = 0.87, see Fig. 5c) than the ones of *C*_*ex*1_ (*R*^2^ = 0.24) or *C*_*ex*2_ (*R*^2^ = 0.42).

Excess commuting can offer valuable insights in evaluating job-housing balance and the overall efficiency of a city. However, the minimized commuting flows are obtained by the global optimum solution, making excess commuting index unable to find local inefficient routes, which could be improved by our PWE method.

### A null model

To explain why most *η*s lie in (0.73, 0.78) (see Table 1), we build a null model to compare the simulation with empirical results. Let us assume a monocentric city, where all the people work at the center of the city and live uniformly around the city space. The road network of the city is in the form of a lattice (e.g. Manhattan grids, see Fig. 2a); therefore, the route distance from the location (*x*, *y*) to the city center (0, 0) is |*x*| + |*y*|, and the geometric distance is (*x*^2^ + *y*^2^)^1/2^. Under these assumptions, we could easily get *η* = 0.77 by simulation (see the Methods). Because the population distribution is not uniform within the city in reality, we also test for normal and exponential population density decay from the city center, which are observed in many cities^39^. The PWEs of the normal, and exponential distributions are 0.79 and 0.80, which implies the concentration at the city center leads to higher efficiency.

The empirical results for monocentric cities in Senegal are well fitted by simulation except for Dakar. In fact, the urban form of Dakar is irregular narrow strip, and cannot be treated as a monocentric city. From both the model and empirical results, we could find that PWE is mainly influenced by the road network structure, and the population distribution. The more direct routes between OD pairs of high traffic volumes, the higher efficiency the road network is. Similarly, increasing the ratio of jobs-housing balance may reduce overall commuting distance and improve the transportation efficiency.

## Discussion

In this paper, basing on CDRs, we introduce a new indicator, the population-weighted efficiency, which tells us how efficient the city is and provides a quantitative measurement to guide transportation infrastructure development. This indicator is helpful for evaluation of urban planning and designing, and could be applied to other spatial networks easily. Besides, our method to obtain the OD matrices is more objective which is based on users’ behavior patterns rather than practical sense. We prove that the PWE is a proper indicator for evaluating the efficiency in two ways. First, according to the PWE, we identify the most inefficient roads both within Dakar and between cities, which are partially verified by ongoing constructed roads. Second, we compare PWE with excess commuting, the most commonly used commuting efficiency index in transportation literature, and find that PWE is highly correlated with the average commuting time, and has a better fitting result than excess commuting.

After comparing the PWE indicator of all ten main cities in Senegal, We discover that Dakar is the most inefficient city, which is mainly due to the lack of fast south-north trending transit and the high degree of work-home separation. Another finding is that most Senegalese cities’ average PWE value are approximately 0.73–0.78, and we believe that this is a reflection of the monocentric feature and Manhattan road networks (i.e., lattice networks), which can be explained by the simple model that we propose. In fact, the efficiency and extendibility of a city is strongly influenced by its terrain, current road network and population distribution, and suitable planning strategies are required for special terrain. For example, Dakar may need to build cross-sea transportation, but another possibility may lead to the redistribution of land use for the historical heart of the city.

In addition, different types of people may tend to have quite different mobility patterns. The mobility dynamics for local and non-local users may not be similar (see Supplementary Fig. 4) as well as for people with different income^24^,^40^. For future studies, it will be critical to distinguish between different dynamics for different types of users^41^, which could be helpful when performing simulations for policy impact evaluations or urban planning and real-estate development. One more possible exploration is assessing the infrastructure development within a region by comparing the infrastructure density (e.g., road density) and population density within the same region (see Supplementary Fig. 4).

## Methods

### OD detection

By integrating the CDR data records, we obtain a list of places a user visited with information of both when and how many times these visits occurred. Then, we count the locations at which the user appears at both night and day and consider the places that appear with the highest frequencies to be the home and workplace. When we fail to detect the work or home location, we assign it as UNKNOWN. If a user’s home is unknown but work is known (or vice verse), we still accumulate the data point as the daytime population and draw it out together in Fig. 6. We obtain an extended OD matrix with an extra row and column for users without a detected home or work place, respectively. There’s no user with both unknown home and work place, but the non-local users in Dakar are easily absent of a home location (see discussion in Supplementary Fig. 3), which also happens in developed countries^37^.

### Road network data and efficiency

The Google Map API (http://maps.googleapis.com/maps/api/) can return the travel time (by car, in seconds) and route distance between two locations, and the geometric distance between these two locations can be easily calculated according to their (longitude, latitude)s. In addition, the Google API can return real-time data on travel time in big cities, which could be used to compute the average commuting time in cities.

### Simulation for the null model

The simulation is performed as follows. We split the entire *L* × *L* space into *L*^2^ lattices, which represent Manhattan road networks. We uniformly generate 100,000 points, which are located at the intersections of the road network. Therefore, we calculate the geometric distance (*x*^2^ + *y*^2^)^1/2^ and route distance (|*x*| + |*y*|) to the center of the *L* × *L* space, where the central lattice represents the working place. The PWEs of the uniform, normal, and exponential distributions are 0.77(0.001), 0.79(0.001), and 0.80(0.001), respectively. Each simulation was performed 10 times under the *R Environment*.

## Additional Information

**How to cite this article**: Dong, L. *et al.* Population-weighted efficiency in transportation networks. *Sci. Rep.* **6**, 26377; doi: 10.1038/srep26377 (2016).

## Supplementary Material

Supplementary Information

## Figures and Tables

**Table 1:** Population, population density, population-weighted commuting efficiency (PWE) and excess commuting index of the ten main cities in Senegal.

City name	Pop.(10^4^)	Den.(*pop*./*km*^2^)	PWE	*C*_*min*_(*km*)	*C*_*act*1_(*km*)	*C*_*act*2_(*km*)	*C*_*ex*1_	*C*_*ex*2_
Dakar	333	5447	0.683	1.28	2.20	3.31	41.8	61.3
Diourbel	22	292	0.732	1.36	1.88	2.57	27.7	47.1
Kaolack	23	1036	0.721	1.64	2.45	3.44	33.1	52.3
Louga	20	181	0.755	1.44	1.76	2.46	18.2	41.5
Mbour	42	680	0.725	1.73	3.03	4.34	42.9	60.1
Saint-Louis	26	735	0.730	1.31	2.44	3.32	46.3	60.5
Thies	36	1333	0.716	1.09	2.00	2.85	45.5	61.8
Tivaouance	12	323	0.777	1.32	1.91	2.50	30.9	47.2
Touba	88	980	0.729	1.46	2.37	3.30	38.4	55.8
Ziguinchor	20	962	0.777	1.09	1.85	2.52	41.1	56.7

The population is based on WorldPop data^35^.
