# Supplementary material for: Population-weighted efficiency in transportation networks
Source: Sci Rep. 2016 May 27;6:26377. doi: 10.1038/srep26377 (PMC4882487; doi:10.1038/srep26377)
Supplement: Supplementary Information [file srep26377-s1.pdf]

Supplementary Information for Population-weighted efficiency in transportation networks

Lei Dong[1], Ruiqi Li*[2], Jiang Zhang†[2], and Zengru Di[2]

[1]School of Architecture, Tsinghua University, Beijing, 100084, China
[2]School of Systems Science, Beijing Normal University, Beijing, 100875, China

Supplementary Figure 1

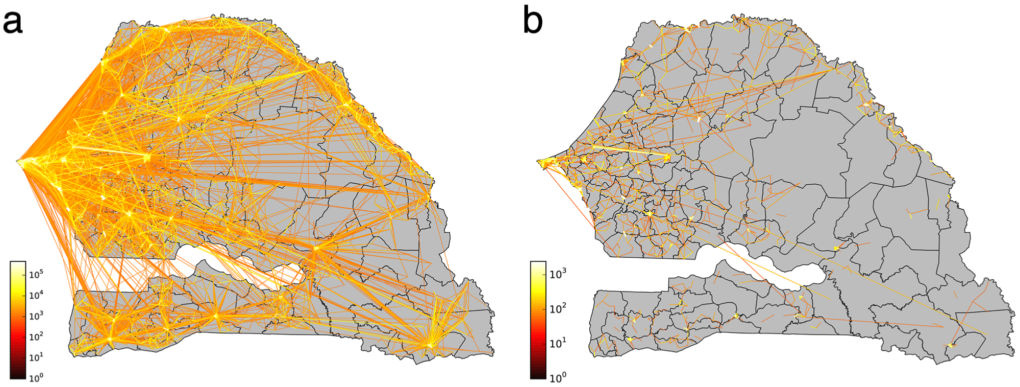

Supplementary Figure 1: At antenna resolution, (a) the cumulative mobility volume of one year (25 fortnights, more accurately) of all the mobile phone users. Once his/her site location is different from previous one and the time interval is not too long (within one day in our research), then we identify it as a direct mobility event. (b) the stable dominant origins-destinations (ODs) pairs extracted from mobility traces with timestamp information (see the Methods in the main text). And there's a significant 'twin-region' on the west-central region of Senegal. For better visualization, we didn't show the pairs whose volume is lower than 100 and 50 for (a) and (b), respectively. Maps were created using Python 2.7 (http://www.python.org).

Supplementary Fig.1(a) is extracted from the CDRs at antenna resolution. If one user firstly appeared in location 'A', and then, within a not too long time interval (one day in our research), appeared in location 'B', we then identify there's a direct movement from 'A' to 'B' (one of inevitable drawback for extracting mobility from the CDRs is that only when the user have an activity then his/her position will be recorded. So there will certainly be some missing of mobility traces). By integrating all the traces of all the users, we can get

*liruiqi@mail.bnu.edu.cn
†zhangjiang@bnu.edu.cn

a mobility network at nation level. This mobility network is highly probably composed by all types of movements. An important question is that among all the movements, what's the proportion of the rigid travel demand (e.g., from home to work and back), which is quite important for efficiency of a city and urban transportation optimization. So in Supplementary Fig. 1(b), we extract the OD network at nation level by the OD detection method we proposed in the main text. An attractive finding is that there is one stable, high-volume OD pair (actually two pairs who are quite close to each other, these regions are within Tivaouane and Touba) spanning long geometric distance in the west-central region of Senegal, which means that there are quite a lot of people who work at one city and reside in another. This phenomenon may reveal the existence of a 'twin-region' who are strongly connected but geometrically a little far away from each other, which may origin from complementary advantages of two cities (or more smaller regions) and the rapid transportation. Actually, due to the population size, the cumulative OD volume is not that high, causing the 'twin-city' phenomenon is not that obvious.

Supplementary Figure 2

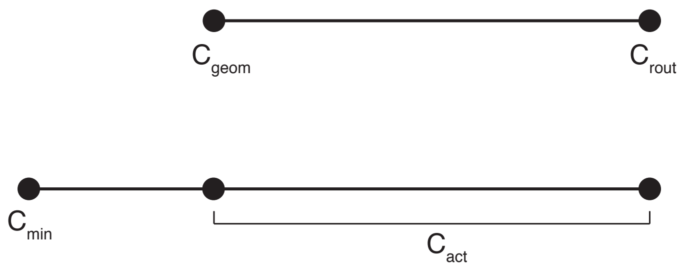

Supplementary Figure 2: Schematic of excess commuting metrics. In the figure below, C_{act} is the actual commuting distance, which is C_{geom} at individual level if measured by geometric distance, and C_{rout} by route distance. C_{min} is the minimum commuting distance when all working and living places are fixed in urban area, workers in a city choose work places that is closed to their living places. $C_{ex} = (C_{act} - C_{min})/C_{min} \times 100$.

Supplementary Figure 3

Supplementary Fig.3 is the density map of different types of users (local and non-local) at different time (day and night). (a) and (b) are local users' distribution at daytime and night, respectively; (c) and (d) are for non-local users. The distribution of non-local people, who mainly distribute at the Dakar Plateau and the airport, is more concentrated than local people. When we distinguish local and non-local users in Dakar, we only consider the appearance frequency as the indicator. If a user spend more than 2/3 of his or her time at Dakar, we identify him or her as a local user of Dakar, and vice versa. Although this will introduce some arbitrariness, it still give us some qualitatively distinguishable results. Besides, this method will be quite useful if we can only get access to one region's CDRs. In the future, we can cross examine the accuracy of this method with nationwide OD information, which may give us a more precise duration threshold to distinguish the local and non-local users in a region.

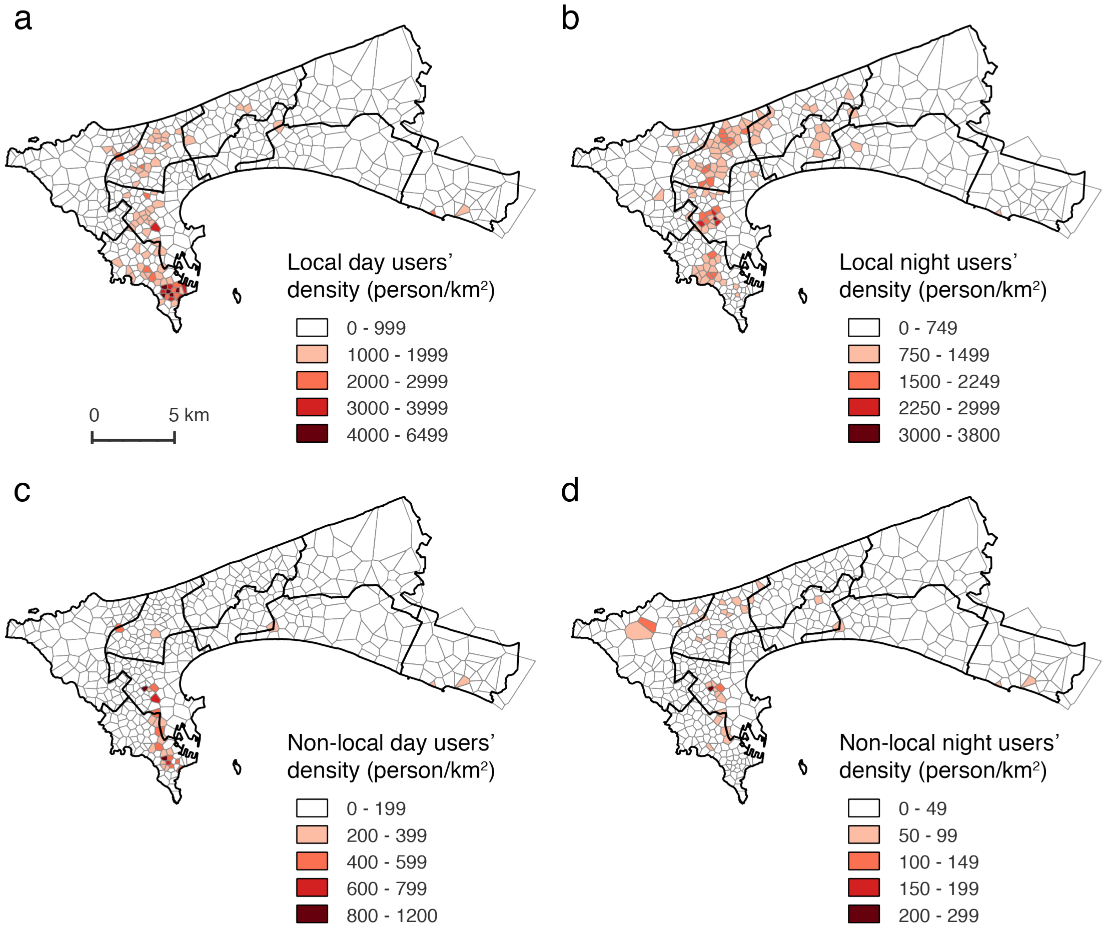

Supplementary Figure 3: Density distribution of (a) local users in the daytime; (b) local users at night; (c) non-local users in the daytime; (d) non-local users at night, the top-left hotspot is Dakar airport. The distribution of non-local people is more concentrated, mainly at the Dakar Plateau and the airport. Maps were created using QGIS 2.8 (http://www.qgis.org).

Here it should be noted that when we try to distinguish local and non-local users. About 170,000 users had appeared in Dakar, so, in principle, we can distinguish all of these users as local and non-local users. But since in this project we mainly concern the commuting efficiency of a city, so we neglect the population whose home and workplace are the same, which corresponds to about 90,000 users. The remaining population size is 87302. According to our criteria, there are 77076 and 10226 local and non-local users, respectively. Among all the 77076 local users, there are 62562 users with complete OD information, while 14371 users are absent of home location and 143 absent of work location. For all the 10226 non-local users, those three numbers are 2286, 7494, 446, respectively. In Supplementary Fig. 3, we only show the distribution of users with complete OD information. An attractive finding is that a large proportion of non-local users (i.e., 7494 out of 10226) are absent of home information, which indicates that they are not active during the night or they leave Dakar in the day. By contrast, the absent proportion for

local users are relatively much smaller. This finding is also backing our hypothesis that the behavioral dynamics for local and non-local users are different.

Supplementary Figure 4

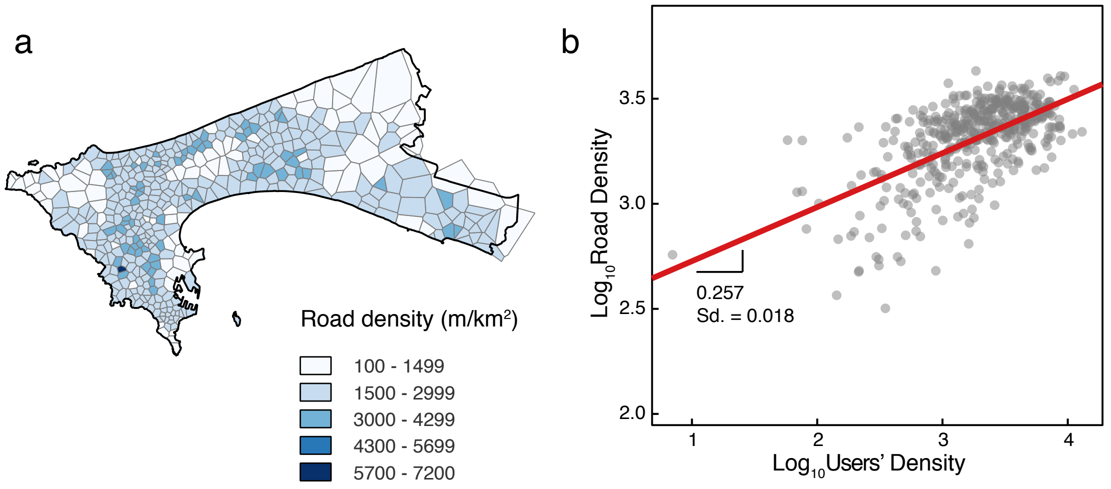

Supplementary Figure 4: (a) Road density ($Road\ length/Area$); (b) scatter plot of users' density (x-axis) and road density (y-axis), the log-log regression result is shown in the figure, the slop is 0.257 (0.018). The Map was created using QGIS 2.8 (http://www.qgis.org).

Supplementary Fig.4(a) is the road density ($Road\ length/Area$) of Dakar at the level of the Voronoi cell, which is generated according to the distribution of antennas; (b) is the scatter plot of users' density (x-axis) and road density (y-axis). We can assess the infrastructure deployment within a region quantitatively by comparing the infrastructure density (here is road density) and the population density within the same region. From the scatter plot of population density and road density, we can identify the regions which are in need of more road constructions (i.e., points far below the red line).

Supplementary Table 1

Top ten inefficient routes calculated by $Eq.$ (2) in the main text. The last two columns are the antenna indexes, which are labeled by the D4D datasets.

Supplementary Table 2

Day-night separation degree of each $Region$ is calculated as follows:

$$\frac{\sum_{i=1}^{n} |ratio_{day_i/night_i} - 1|}{n}, \tag{1}$$

where i means the ith number of $Department$, and n means the total number of $Department$. $Ratio_{day_i/night_i}$ equals the day time population divided by night time population. The separation degree of each $Region$ is shown in Supplementary Tab. 2 (It

d_e/d_r	#Commuters	#From	#To
0.248	76	380	367
0.265	73	261	271
0.301	62	374	367
0.395	88	360	373
0.397	72	117	100
0.403	57	391	377
0.448	124	154	162
0.459	55	154	135
0.461	115	57	58
0.463	56	57	72

Supplementary Table 1: Top ten inefficient routes.

should be noted that here, *Department* and *Region* are all Senegal administrative divisions, which are different from the definition of the cities that we used above.)

Region name	Separation degree
Dakar	0.079
Diourbel	0.038
Fatick	0.051
Kaffrine	0.031
Kaolack	0.064
Kedougou	0.010
Kolda	0.022
Louga	0.061
Matam	0.020
Saint-Louis	0.039
Sedhiou	0.029
Tambacounda	0.007
Thies	0.044
Ziguinchor	0.026

Supplementary Table 2: Day-night (or called work-home) separation degree of *Regions*.
